# Bisphenol S and F: A Systematic Review and Comparison of the Hormonal Activity of Bisphenol A Substitutes

**DOI:** 10.1289/ehp.1408989

**Published:** 2015-03-16

**Authors:** Johanna R. Rochester, Ashley L. Bolden

**Affiliations:** The Endocrine Disruption Exchange (TEDX), Paonia, Colorado, USA

## Abstract

**Background:**

Increasing concern over bisphenol A (BPA) as an endocrine-disrupting chemical and its possible effects on human health have prompted the removal of BPA from consumer products, often labeled “BPA-free.” Some of the chemical replacements, however, are also bisphenols and may have similar physiological effects in organisms. Bisphenol S (BPS) and bisphenol F (BPF) are two such BPA substitutes.

**Objectives:**

This review was carried out to evaluate the physiological effects and endocrine activities of the BPA substitutes BPS and BPF. Further, we compared the hormonal potency of BPS and BPF to that of BPA.

**Methods:**

We conducted a systematic review based on the Office of Health Assessment and Translation (OHAT) protocol.

**Results:**

We identified the body of literature to date, consisting of 32 studies (25 *in vitro* only, and 7 *in vivo*). The majority of these studies examined the hormonal activities of BPS and BPF and found their potency to be in the same order of magnitude and of similar action as BPA (estrogenic, antiestrogenic, androgenic, and antiandrogenic) *in vitro* and *in vivo*. BPS also has potencies similar to that of estradiol in membrane-mediated pathways, which are important for cellular actions such as proliferation, differentiation, and death. BPS and BPF also showed other effects *in vitro* and *in vivo*, such as altered organ weights, reproductive end points, and enzyme expression.

**Conclusions:**

Based on the current literature, BPS and BPF are as hormonally active as BPA, and they have endocrine-disrupting effects.

**Citation:**

Rochester JR, Bolden AL. 2015. Bisphenol S and F: a systematic review and comparison of the hormonal activity of bisphenol A substitutes. Environ Health Perspect 123:643–650; http://dx.doi.org/10.1289/ehp.1408989

## Introduction

There is increasing evidence that bisphenol A (BPA)—used in plastics, receipts, food packaging, and other products—might be harmful to human health due to its actions as an endocrine-disrupting chemical (EDC) (Bonefeld-Jørgensen et al. 2007; Richter et al. 2007b; Rochester 2013). Scientists, regulators, and the general public have raised concerns about the use of BPA, especially because of its ubiquitous nature and potential for continuous exposure (Vandenberg et al. 2010). This has prompted industry to seek alternative chemicals. As manufacturers have begun to remove BPA from their products as a result of consumer concern, there has been a gradual shift to using bisphenol analogs. For the purpose of our review, we chose to evaluate two of these analogs—bisphenol S (BPS) and bisphenol F (BPF)—because of their widespread consumer and commercial use. BPS is used for a variety of industrial applications, for example, as a wash fastening agent in cleaning products, an electroplating solvent, and a constituent of phenolic resin (Clark 2012). BPS is also used as a developer in thermal paper, including products marketed as “BPA-free paper” (Liao et al. 2012c). BPF is used to make epoxy resins and coatings, especially for systems needing increased thickness and durability (i.e., high-solid/high-build systems), such as tank and pipe linings, industrial floors, road and bridge deck toppings, structural adhesives, grouts, coatings, and electrical varnishes (Fiege et al. 2000). BPF epoxy resins are also used for several consumer products such as lacquers, varnishes, liners, adhesives, plastics, water pipes, dental sealants, and food packaging (Office of Environmental Health Hazard Assessment 2012). BPS and BPF have been detected in many everyday products, such as personal care products (e.g., body wash, hair care products, makeup, lotions, toothpaste) (Liao and Kannan 2014), paper products (e.g., currency, flyers, tickets, mailing envelopes, airplane boarding passes) (Liao et al. 2012c), and food (e.g., dairy products, meat and meat products, vegetables, canned foods, cereals) (Liao and Kannan 2013). BPS, BPF, and BPA have been detected in indoor dust at the following concentrations: BPS, 0.34 μg/g; BPF, 0.054 μg/g; BPA, 1.33 μg/g (Liao et al. 2012b). BPS and BPF have also been detected in surface water, sediment, and sewage effluent, generally at lower concentrations than BPA, but in the same order of magnitude (Fromme et al. 2002; Song et al. 2014; Yang et al. 2014). In humans, BPS and BPF have been detected in urine at concentrations and frequencies comparable to BPA (Liao et al. 2012a; Zhou et al. 2014). In urine samples from 100 American, nonoccupationally exposed adults, Liao et al. (2012a) found BPF in 55% of samples at concentrations up to 212 ng/mL, and BPS in 78% of samples at concentrations up to 12.3 ng/mL. BPA was found in 95% of the samples, with concentrations up to 37.7 ng/mL.

**Figure 1:**
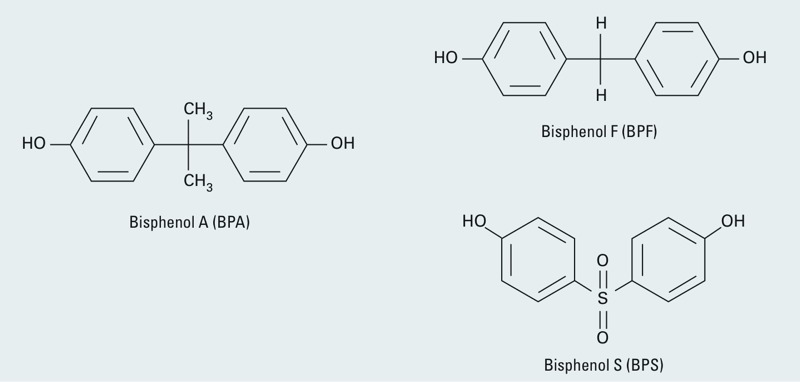
Chemical structures of bisphenol A, bisphenol S, and bisphenol F.

Ideally, substitutes used to replace a chemical of concern would be inert, or at least far less toxic than the original chemical(s). Unfortunately, many chemical replacements are untested before being placed on the market, and in some cases are similar enough to the original chemical to cause concern. For that reason, such chemical analogs should be evaluated before they are used as replacements for toxic chemicals. These chemicals may be just as harmful as the originals—or more so—and have been described as “regrettable substitutions,” as is the case with several perfluorinated chemicals (Howard 2014), pesticides (Coggon 2002), and flame retardants (Bergman et al. 2012). In the case of BPS and BPF, these chemicals are structural analogs to BPA (Figure 1); thus their effects in physiological systems may be similar. BPA is a known endocrine disruptor based on *in vitro* (Wetherill et al. 2007) and animal laboratory studies (Richter et al. 2007a; Vandenberg 2014b), and exposures to environmental levels of BPA have been associated with adverse health outcomes in children and adults in more than 75 human studies (Rochester 2013). To evaluate the endocrine-disrupting properties of the BPA substitutes BPS and BPF, we conducted a systematic review of the literature using the National Institute of Environmental Health Sciences’ Office of Health Assessment and Translation (OHAT) systematic review protocol (National Toxicology Program 2013; Rooney et al. 2014). In this analysis we summarize *in vivo* and *in vitro* literature and compare the hormonal potency of BPS and BPF to BPA using the *in vitro* studies.

*Literature search and review*. We performed a comprehensive literature search in order to identify studies describing endocrine and other physiological effects of exposure to BPF and BPS. The search included all articles published and indexed for all years to June 2014. Electronic searches were performed in Web of Science (https://webofknowledge.com/) and PubMed (http://www.ncbi.nlm.nih.gov/pubmed) using CAS registry numbers and common names. Our search logic is summarized in Table 1.

**Table 1:** BPS and BPF search logic.

	PubMed and Web of Science search logic
BPF	620-92-8[EC/RN] OR bisphenol-F OR (bisphenol* AND BPF) OR bis(4-hydroxyphenyl)methane OR bis(p-hydroxyphenyl)methane OR bis(4-hydroxyphenyl)-methane OR bis(p-hydroxyphenyl)-methane OR p-(p-hydroxybenzyl)phenol OR p-(p-hydroxybenzyl)-phenol OR 4-(4-hydroxybenzyl)phenol OR 4-(4-hydroxybenzyl)-phenol OR “4,4’-methylenebis(phenol)” OR “p,p’-bis(hydroxyphenyl)methane” OR “p,p’-bis(hydroxyphenyl)-methane” OR “4,4’-bis(hydroxyphenyl)methane” OR “4,4’-bis(hydroxyphenyl)-methane” OR “4,4’-dihydroxydiphenylmethane” OR “4,4’-dihydroxydiphenyl-methane” OR “4,4’-methylenediphenol” OR “4,4’-methylene-diphenol” OR “4,4’-methylenebisphenol” OR “4,4’-methylene-bisphenol”
BPS	80-09-1[EC/RN] OR bisphenol-S OR [(bisphenol OR bisphenols) AND BPS] OR bis(4-hydroxyphenyl)-sulfone OR bis(4-hydroxyphenyl)sulfone OR bis(4-hydroxyphenyl)-sulphone OR bis(4-hydroxyphenyl)sulphone OR bis(p-hydroxyphenyl)-sulfone OR bis(p-hydroxyphenyl)sulfone OR bis(p-hydroxyphenyl)-sulphone OR bis(phydroxyphenyl)sulphone OR 4,4’-dihydroxydiphenyl-sulfone OR 4,4’-dihydroxydiphenylsulfone OR 4,4’-dihydroxydiphenyl-sulphone OR 4,4’-dihydroxydiphenylsulphone OR p,p’-dihydroxydiphenyl-sulfone OR p,p’-dihydroxydiphenylsulfone OR p,p’-dihydroxydiphenyl-sulphone OR p,p’-dihydroxydiphenylsulphone OR 4,4’-sulfonyldiphenol OR 4,4’-sulfphonyldiphenol OR p,p’-sulfonyldiphenol OR p,p-sulfphonyldiphenol OR 4,4’-sulfonylbisphenol OR 4,4’-sulfphonylbisphenol OR p,p’-sulfonylbisphenol OR p,p-sulfphonylbisphenol OR 4,4’-sulfonylbiphenol OR 4,4’-sulfphonylbiphenol OR p,p’-sulfonylbiphenol OR p,p’-sulfphonylbiphenol OR 4-hydroxyphenyl-sulfone OR 4-hydroxyphenylsulfone OR 4-hydroxyphenyl-sulphone OR 4-hydroxyphenylsulphone OR p-hydroxyphenyl-sulfone OR p-hydroxyphenylsulfone OR p-hydroxyphenyl-sulphone OR p-hydroxyphenylsulphone

For inclusion, the studies had to be primary literature and assess any *in vitro* or *in vivo* physiological effects of BPS or BPF exposure. Two independent reviewers (J.R.R. and A.L.B.) screened all titles and abstracts for relevancy, using Distiller SR® software (Evidence Partners), and resolved any conflicts or discrepancies. Data from the studies were extracted, and were cross-checked by the two reviewers. When needed, data were extracted from figures or graphs using Universal Desktop Ruler® software (version 3.6; AVPSoft), with measurements taken in triplicate by a single reviewer.

**Figure 2:**
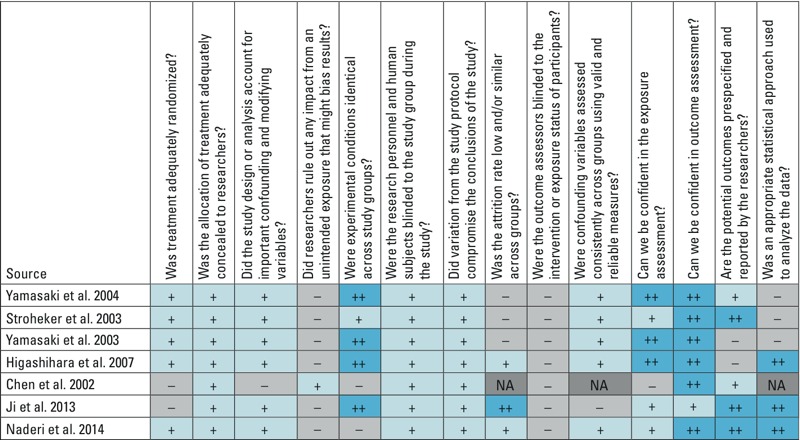
Risk of bias (RoB) ratings for BPS and BPF *in vivo* studies. Abbreviations: ^++^, definitely low risk of bias; ^+^, probably low risk of bias; –, probably high risk of bias; NA, not applicable.

Study quality for *in vivo* studies was assessed using a protocol developed by OHAT. Briefly, risk of bias (RoB) in experimental methodology was assessed by answering 14 questions. The RoB questions covered biases in subject selection, protocol performance, attrition/exclusion of subjects, detection of outcomes, selective reporting of outcomes, and statistical methodology. Questions were rated as “definitely low RoB,” “probably low RoB,” “probably high RoB,” or “definitely high RoB” depending on standardized responses. The individual RoB questions are provided in Figure 2. Next, “key” study quality questions, identified *a priori*, were used to determine the initial quality of each study, then ratings of the remaining questions were used to determine the overall study quality: “low,” “moderate,” or “high.” If any study received a “low” rating, it was removed from analysis. This protocol has been described in detail elsewhere (National Toxicology Program 2013; Rooney et al. 2014).

As specified in the OHAT protocol (National Toxicology Program 2013; Rooney et al. 2014), *in vitro* studies were not assessed for quality, but were used to support specific *in vivo* end points. For example, estrogen receptor (ER) binding or activation studies support the biological plausibility of increased uterine growth, an *in vivo* estrogenic response. Where there were at least three *in vitro* studies, the strength of support was rated on the following factors: relevance of biological process or pathway to human disease, consistency across model systems (where there were more than two systems), physiological relevance of the dose concentration, potency (magnitude of response compared with positive control), dose response (monotonic or nonmonotonic), and publication bias. These factors were integrated for a final rating of “weak,” “moderate,” or “strong” *in vitro* support of the biological plausibility of *in vivo* observations, but they were not used to exclude studies. *In vitro* observations that had fewer than three studies per end point, or did not relate to any observed *in vivo* end points, are described in the text.

## Results

Our search identified 1,370 studies; of these, 32 studies (25 *in vitro* only and 7 *in vivo*) were identified as relevant for inclusion. Figure 2 shows the study quality ratings for the *in vivo* studies. All studies were rated moderate quality or better; therefore, no *in vivo* studies were removed because of low quality.

*BPS*. The literature reporting the physiological effects of BPS exposure consisted of 4 *in vivo* studies and 18 *in vitro* studies. The *in vivo* studies are presented in Table 2. BPS exposure caused acute toxicity in *Daphnia magna* (Chen et al. 2002). Yamasaki et al. (2004) found that postnatal BPS exposure in rats caused an induction of uterine growth, a marker of estrogen exposure (Owens and Ashby 2002), at the lowest and highest doses. The authors also found that BPS bound to the nuclear ER at 0.0055% relative binding affinity (Yamasaki et al. 2004). Ji et al. (2013) studied BPS exposure in zebrafish (*Danio rerio*) and found decreases in gonad weight, alterations in plasma estrogen and testosterone, and disrupted reproduction (i.e., decreased egg production and hatchability, increased time to hatch, increased embryo malformations). Another study in zebrafish showed that BPS exposure increased female to male sex ratio; decreased body length; altered testosterone, estradiol, and vitellogenin concentrations; and led to reproductive disruption (i.e., decreased egg production, increased time to hatch, decreased sperm count) (Naderi et al. 2014).

**Table 2:** *In vivo* BPS and BPF hormonal/physiological effect studies.

Chemical	Study	Model	Exposure duration	Age at exposure	Route of exposure	Doses	LOEL^*a*^	Results
BPS	Chen et al. 2002	*Daphnia magna*	2 or 4 days	Juvenile	Culture	NA	NA	BPS was acutely toxic in *Daphnia magna;* EC_50_, 76 mg/L (24 hr); EC_50_ 55 mg/L (48 hr). BPS showed estrogenic activity and did not show mutagenic activity *in vitro*.
BPS	Yamasaki et al. 2004	Rat	3 days	20 days	Injection	0, 20, 100, 500 mg/kg/day	20 mg/kg	BPS exposure was estrogenic in rats via increases in uterine weight. BPS was also found to bind the estrogen receptor.
BPS	Ji et al. 2013	*Danio rerio*	21 days	3–5 months	Water	0, 0.5, 5, 50 μg/L	0.5 μg/L	BPS exposure in zebrafish showed decreases in gonad weight with respect to body weight in males and females. No changes were observed in liver or brain weight with respect to body weight. E_2_ levels were increased in males and in females, T levels were decreased in males, and E_2_/T ratios were increased in males and females. Reproduction was impaired as evidenced by decreased egg production and hatchability, and by increased time to hatch and embryo malformation rates. Gene expression in the brain and gonads of several genes involved in the hypothalamic–pituitary–gonadal axis were altered in males and females.
BPS	Naderi et al. 2014	*Danio rerio*	75 days	4–6 months	Water	0, 0.1, 1, 10, 100 μg/L	1 μg/L	BPS exposure in zebrafish showed decreased body length and weight in males, increased female to male sex ratio, decreased gonad weight, increased liver weight, decreased T_3_ and T_4_, decreased T in males, increased E_2_ in males and females, and increased VTG in males and females. BPS also caused disrupted reproduction, with decreased number of eggs produced, decreased hatching rate, increased time to hatch, and decreased sperm count.
BPF	Chen et al. 2002	*Daphnia magna*	2 or 4 days	Juvenile	Culture	NA	NA	EC_50_, 80 mg/L (24 hr); and EC_50_ 56 mg/L (48 hr). BPF showed estrogenic activity and did not show mutagenic activity *in vitro*.
BPF	Yamasaki et al. 2003	Rat	10 days	19 days	Gavage	0, 50, 200, 1,000 mg/kg/day	100 mg/kg	BPF co-administered with TP increased the weight of the Cowper’s gland. BPF alone and combined with TP decreased body weight.
BPF	Yamasaki et al. 2004	Rat	3 days	20 days	Injection	0, 100, 300, 1,000 mg/kg/day	100 mg/kg	BPF induced uterine growth in immature rats. BPF was positive for relative binding affinity (E_2_).
BPF	Higashihara et al. 2007	Rat	28 days	8 weeks	Gavage	0, 20, 100, 500 mg/kg/day	20 mg/kg	There were decreases in body weight and food consumption in males and females treated with BPF. Hematological and biochemical parameters were altered, including decreased cholesterol and glucose in males and females. BPF treatment decreased T_3_ and increased T_4_ levels. BPF increased testes, liver, thyroid, brain, and kidney weights.
BPF	Stroheker et al. 2003	Rat	4 days	22 days	Gavage	0, 25, 50, 100, 200 mg/kg/day	100 mg/kg	BPF was shown to increase uterine weight in rats.
Abbreviations: EC_50_, half-maximal effective concentration; NA, not available; T, testosterone; T_3_, triiodothyronine; T_4_, thyroxin; TP, testosterone propionate; VTG, vitellogenin. ^***a***^The dose at the end point of the lowest observed effect.								

*In vitro* data from 12 studies assessing estrogenicity provided strong evidence supporting the estrogenic responses observed in *in vivo* studies (Table 3), based on relevance of the end point to human health [e.g., interaction with human ERα and G-protein coupled receptor 30 (GPR30)], consistent response across eight cell lines, and physiologically relevant concentrations assessed (micromolar range) (Chen et al. 2002; Grignard et al. 2012; Hashimoto and Nakamura 2000; Hashimoto et al. 2001; Kitamura et al. 2005; Kuruto-Niwa et al. 2005; Molina-Molina et al. 2013; Rajasärkkä et al. 2014; Rosenmai et al. 2014; Teng et al. 2013; Viñas and Watson 2013a, 2013b). Several of these studies showed that BPS had weaker estrogenic potency than estradiol (E_2_) when assayed in nuclear receptor models (Chen et al. 2002; Grignard et al. 2012; Hashimoto and Nakamura 2000; Hashimoto et al. 2001; Kitamura et al. 2005; Kuruto-Niwa et al. 2005; Molina-Molina et al. 2013; Teng et al. 2013). However, two studies (Viñas and Watson 2013a, 2013b) showed that BPS had equivalent or greater estrogenic potency to E_2_ when assayed in membrane receptor models; BPS induced membrane receptor–mediated pathways typically up-regulated by E_2_. Four studies showed that BPS bound to the ER in competitive binding assays (Grignard et al. 2012; Hashimoto et al. 2001; Molina-Molina et al. 2013; Yamasaki et al. 2004). There was also one study showing androgenic activity of BPS (Molina-Molina et al. 2013) and one study showing antiandrogenic activity (Kitamura et al. 2005). In addition, in other *in vitro* experiments BPS exposure induced caspase 8 production, which indicates that BPS may alter cellular apoptotic and survival signaling (Salvesen and Walsh 2014; Viñas and Watson 2013a, 2013b). BPS also had effects on hepatic cells (Peyre et al. 2014); it bound to serum albumins (Mathew et al. 2014), and it caused DNA damage (Fic et al. 2013; Hashimoto and Nakamura 2000; Lee et al. 2013).

**Table 3:** Studies assessing BPS and BPF activity *in vitro*.

Study	Chemical(s) tested	End point measured	Concentrations tested
Audebert et al. 2011	BPF	Cytotoxicity, genotoxicity	1 to 100 μM
Cabaton et al. 2006	BPF/BPS	Antiandrogenicity, estrogenicity, genotoxicity	10^–11^ to 10^–5^ M and 36.4 to 170 μM
Chen et al. 2002	BPF/BPS	Acute toxicity, estrogenicity	0.01 to 100 mg/L
Fic et al. 2013	BPF/BPS	Cytotoxicity, genotoxicity, mutagenicity	12.5 to 100 μM, 0.1 to 10 μM, and 4 to 500 μg/plate
Grignard et al. 2012	BPS	Estrogenicity	10^–12^ to 10^–4^ M
Hashimoto and Nakamura 2000	BPF/BPS	Estrogenicity	10^–7^ to 10^–3^ M
Hashimoto et al. 2001	BPF/BPS	Estrogenicity	10^–9^ to 10^–3^ M
Kidani et al. 2010	BPF	Adiponectin	80 μM
Kitamura et al. 2003	BPF	Estrogenic, estrogen CBA	10^–8^ to 10^–4^ M
Kitamura et al. 2005	BPF/BPS	Antiandrogenicity, estrogenicity	10^–7^ to 10^–4^ M
Kuruto-Niwa et al. 2005	BPS	Estrogenicity	10^–7^ to 10^–4^ M
Lee et al. 2013	BPF/BPS	Cytotoxicity, genotoxicity	10 to 250 μM
Mathew et al. 2014	BPS	Serum albumin binding	0.2 to 4 μM
Molina-Molina et al. 2013	BPF/BPS	Androgenicity, antiandrogenicity, estrogenicity, estrogen CBA	10^–8^ to 10^–5^ M
Nakagawa and Tayama 2000	BPF	Cytotoxicity, mitochondrial function	0.25 to 1 mM
Ogawa et al. 2006	BPF	Estrogenicity	10^–7^ to 10^–3^ M
Perez et al. 1998	BPF	Estrogenicity	10^–8^ to 10^–5^ M
Peyre et al. 2014	BPS	Hepatic cell function	1 to 500 μM
Pisapia et al. 2012	BPF	Estrogenicity	10^–7^ to 10^–5^ M
Rajasärkkä et al. 2014	BPF/BPS	BPA activity, estrogenicity	10^–7^ to 10^–2^ M
Rosenmai et al. 2014	BPF/BPS	Antiandrogenicity, estrogenicity, steroidogenesis, AhR activity	10^–4^ to 10^2^ μM
Satoh et al. 2004	BPF	Antiandrogenicity, cytotoxicity, estrogenicity, estrogen and androgen CBA	10^–9^ to 10^–3^ M
Stroheker et al. 2004	BPF	Antiandrogenicity, antiestrogenicity, estrogenicity, estrogen CBA	10^–10^ to 10^–5^ M
Teng et al. 2013	BPS	Androgenicity, estrogenicity	10^–13^ to 10^–4^ M
Viñas and Watson 2013a	BPS	Estrogenicity	10^–15^ to 10^–7^ M
Viñas and Watson 2013b	BPS	Estrogenicity	10^–14^ M
Yamasaki et al. 2004	BPS	Estrogen CBA	10^–11^ to 10^–4^ M
CBA, competitive binding assay.			

*BPF*. Of the five *in vivo* studies, four showed that BPF was estrogenic, androgenic, and thyroidogenic (Table 2). Nineteen *in vitro* studies showed estrogenic, androgenic, and other physiological/biochemical effects (Table 3). BPF was acutely toxic in *Daphnia magna* (Chen et al. 2002). Two studies showed that BPF exposure induced uterine growth in rats, indicating estrogenic activity (Stroheker et al. 2003; Yamasaki et al. 2004). There were also two studies that showed evidence of androgenic activity: One study indicated that BPF increased the weight of the testes (Higashihara et al. 2007), and the other showed a cumulative effect of BPF when co-administered with testosterone propionate that increased Cowper’s gland weight (Yamasaki et al. 2003). The cumulative effect indicates that BPF may augment other androgens, if indeed it acts synergistically. BPF exposure also increased thyroid weight and altered thyroid hormone concentrations, as well as caused changes to hematological parameters and enzyme expression (Higashihara et al. 2007).

As shown in Table 3, *in vitro* data from 12 studies provided strong evidence that BPF had estrogenic activity, supporting *in vivo* observations. This rating was based on relevance to human health (MCF-7 cell and human ER), consistency across five cell models, and the use of relevant concentrations (micromolar range) (Cabaton et al. 2009; Chen et al. 2002; Hashimoto and Nakamura 2000; Hashimoto et al. 2001; Kitamura et al. 2003, 2005; Molina-Molina et al. 2013; Perez et al. 1998; Pisapia et al. 2012; Rajasärkkä et al. 2014; Rosenmai et al. 2014; Satoh et al. 2004). One study showed that BPF was not estrogenic in a yeast two-hybrid assay (Ogawa et al. 2006). One study indicated that BPF was antiestrogenic (Stroheker et al. 2004). Moderate evidence from 6 studies showed that BPF was antiandrogenic based on relevance to human health [i.e., human androgen receptor (AR)], consistency across four cell models, and potency (i.e., within 100 orders of magnitude of positive control) (Cabaton et al. 2009; Kitamura et al. 2005; Molina-Molina et al. 2013; Rosenmai et al. 2014; Satoh et al. 2004; Stroheker et al. 2004). BPF also showed other *in vitro* effects such as cytotoxicity, cellular dysfunction, DNA damage, and chromosomal aberrations (Audebert et al. 2011; Cabaton et al. 2009; Lee et al. 2013; Nakagawa and Tayama 2000; Pisapia et al. 2012), and decreased adiponectin production and secretion *in vitro* (Kidani et al. 2010).

*Potency of BPS and BPF compared with BPA*. BPS and BPF are already being used as alternatives for BPA; thus, it is important to understand whether these substitutes possess endocrine-disruptive/active properties similar to those of BPA. Seventeen studies tested BPS and/or BPF along with BPA in the same assays, allowing the potencies and mechanisms of action to be directly compared. Table 4 presents these results, comparing the hormonal potencies of BPF and/or BPS to BPA. The average estrogenic potency (mean ± SD) for BPF compared with BPA was 1.07 ± 1.20, with a range of 0.10–4.83. The average estrogenic potency for BPS compared with BPA was 0.32 ± 0.28, with a range of 0.01–0.90. These results indicate that the potencies of BPS and BPF are in the same order of magnitude as the potency of BPA, and BPF may be just as potent (or more potent) than BPA. Further, BPS and BPF have potencies in the same order of magnitude as BPA in regard to androgenic, antiandrogenic, antiestrogenic, and aryl hydrocarbon activity and inhibitory hormonal signaling in adipocytes (Table 4).

**Table 4:** *In vitro* BPS and BPF hormonal activity compared with BPA.

Assay (receptor tested)	Chemical potency vs. positive control (control)	BPA potency vs. positive control (control)	Chemical potency compared with BPA potency^*a*^	Reference
BPS, estrogenic activity
MCF-7 GFP (ERα)	5.54 × 10^–6^ (E_2_)	8.86 × 10^–6^ (E_2_)	0.62	Kuruto-Niwa et al. 2005
E-screen (ERα)	NA (E_2_)	NA (E_2_)	0.67	Hashimoto and Nakamura 2000
Yeast 2-hybrid (ERα)	4.33 × 10^–6^ (E_2_)	2.76 × 10^–5^ (E_2_)	0.16	Hashimoto and Nakamura 2000
E-screen (ERα)	NA (E_2_)	NA (E_2_)	0.90	Hashimoto et al. 2001
Yeast 2-hybrid (ERα)	4.83 × 10^–6^ (E_2_)	2.40 × 10^–5^ (E_2_)	0.20	Hashimoto et al. 2001
Yeast 2-hybrid (ERα)	NC (E_2_)	NC (E_2_)	0.10	Chen et al. 2002
MCF-7 luc (ERα)	7.82 × 10^–6^ (E_2_)	1.37 × 10^–5^ (E_2_)	0.57	Kitamura et al. 2005
MELN (ERα)	9.76 × 10^–6^ (E_2_)	1.77 × 10^–5^ (E_2_)	0.55	Grignard et al. 2012
BG1Luc4E2 (ERα, ERβ)	2.52 × 10^–7^ (E_2_)	3.14 × 10^–6^ (E_2_)	0.08	Grignard et al. 2012
E-screen (ERα)	1.0 × 10^–6^ (E_2_)	3.75 × 10^–5^ (E_2_)	0.03	Molina-Molina et al. 2013
MELN (ERα)	NR	NR	0.04	Molina-Molina et al. 2013
HELN (ERα)	NR	NR	0.10	Molina-Molina et al. 2013
HELN (ERβ)	NR	NR	0.30	Molina-Molina et al. 2013
CV-1 luc (ERα)	5.73 × 10^–5^ (E_2_)	4.63× 10^–4^ (E_2_)	0.12	Teng et al. 2013
GH3/B6/F10 ERK (mER)	0.68 (E_2_)	1.56 (E_2_)	0.43	Viñas and Watson 2013a
GH3/B6/F10 ERK (mER)	1.36 (E_2_)	1.91 (E_2_)	0.71	Viñas and Watson 2013b
Yeast bioreporter (ERα)	NR	NR	0.01	Rajasärkkä et al. 2014
BG1Luc4E2 (ERα)	NC (E_2_)	NC (E_2_)	0.23	Rosenmai et al. 2014
BPS average estrogenic potency compared with BPA (mean ± SD)			0.32 ± 0.28
BPS, antiandrogenic activity
NIH353 + DHT (AR)	0.18 (Flutamide)	0.58 (Flutamide)	0.25	Kitamura et al. 2005
BPS, androgenic activity
MCF-7 AR1 (AR)	9.00 × 10^–7^ (R1881)	2.25 × 10^–6^ (R1881)	0.40	Molina-Molina et al. 2013
PALM (AR)	NR	NR	0.79	Molina-Molina et al. 2013
BPS, BPA activity
Yeast bioreporter (BPAR)	2.50 × 10^–2^ (BPA)	1.00 (BPA)	0.03	Rajasärkkä et al. 2014
BPF, estrogenic activity
E-screen (ERα)	1.0 × 10^–3^ (E_2_)	0.01 (E_2_)	0.10	Perez et al. 1998
E-screen (ERα)	NA (E_2_)	NA (E_2_)	0.89	Hashimoto and Nakamura 2000
Yeast 2-hybrid (ERα)	6.69 × 10^–6^ (E_2_)	2.76 × 10^–5^ (E_2_)	2.42	Hashimoto and Nakamura 2000
E-screen (ERα)	NA (E_2_)	NA (E_2_)	0.99	Hashimoto et al. 2001
Yeast 2-hybrid (ERα)	6.39 × 10^–5^ (E_2_)	2.40 × 10^–5^ (E_2_)	2.67	Hashimoto et al. 2001
Yeast 2-hybrid (ERα)	NC (E_2_)	NC (E_2_)	0.79	Chen et al. 2002
E-screen (ERα)	5.31 × 10^–5^ (E_2_)	1.10 × 10^–5^ (E_2_)	4.83	Stroheker et al. 2004
E-screen (ERα)	4.67 × 10^–6^ (E_2_)	7.78 × 10^–6^ (E_2_)	0.60	Satoh et al. 2004
MVLN luc (ERα)	5.86 × 10^–6^ (E_2_)	1.17 × 10^–5^ (E_2_)	0.50	Satoh et al. 2004
MCF-7 luc (ERα)	8.6 × 10^–6^ (E_2_)	1.37 × 10^–5^ (E_2_)	0.63	Kitamura et al. 2005
E-screen (ERα)	0.55 (E_2_)	0.86 (E_2_)	0.64	Pisapia et al. 2012
E-screen (ERα)	1.0 × 10^–5^ (E_2_)	3.75 × 10^–5^ (E_2_)	0.27	Rajasärkkä et al. 2014
MELN (ERα)	NR	NR	0.48	Molina-Molina et al. 2013
HELN (ERα)	NR	NR	0.29	Molina-Molina et al. 2013
HELN (ERβ)	NR	NR	0.36	Molina-Molina et al. 2013
Yeast bioreporter (ERα)	NR	NR	1	Rajasärkkä et al. 2014
BG1Luc4E2 (ERα)	NC (E_2_)	NC (E_2_)	0.81	Rosenmai et al. 2014
BPF average estrogenic potency compared with BPA (mean ± SD)			1.07 ± 1.20
BPF, antiandrogenic activity
MDA-MB453+DHT (AR)	NR	NR	0.78	Stroheker et al. 2004
AR-EcoScreen+DHT (AR)	0.03 (Cyproterone acetate)	0.06 (Cyproterone acetate)	0.52	Satoh et al. 2004
NIH353+DHT (AR)	0.21 (Flutamide)	0.58 (Flutamide)	0.36	Kitamura et al. 2005
PALM (AR)	NR	NR	0.13	Molina-Molina et al. 2013
CHO AR (AR)	NC (R1881)	NC (R1881)	0.94	Rosenmai et al. 2014
BPF average antiandrogenic potency compared with BPA (mean ± SD)			0.55 ± 0.32
BPF, antiestrogenic activity
E-screen+tamoxifin (ERα)	NR	NR	1.12	Stroheker et al. 2004
BPF, adiponectin secretion
3T3-L1	NR	NR	0.56	Kidani et al. 2010
BPF, BPA activity
Yeast bioreporter (BPAR)	2.50 × 10^–3^ (BPA)	1.00 (BPA)	0.003	Rajasärkkä et al. 2014
BPF, AhR activity
H4IIE/CALUX (AhR)	NC (TCDD)	NC (TCDD)	1.2	Rosenmai et al. 2014
Abbreviations: AhR, aryl hydrocarbon receptor; AR, androgen receptor; BPAR, BPA-targeted receptor; DHT, dihydrotestosterone; GFP, green fluorescent protein; luc, luciferase; mER, membrane estrogen receptor; NA, not available; NC, not able to calculate from the data presented (e.g., the positive control values were not reported); NR, not reported; TCDD, 2,3,7,8-tetrachlorodibenzo-*p*-dioxin. ^***a***^Potencies were calculated by dividing the BPS or BPF potency by the BPA potency in the same study.				

Rosenmai et al. (2014) used several assays to assess steroidogenic activity, as well as teratogenicity, genotoxicity, carcinogenicity, and metabolic effects. Similar to the present evaluation, they found that BPS and BPF had estrogen receptor binding, estrogenic activity, and antiandrogenic activity similar to those of BPA, with BPS being the least potent. However, BPS and BPF exhibited the greatest steroidogenic (i.e., progesterone) activity, increasing levels of 17α-hydroxyprogesterone and progesterone levels, whereas BPA did not (Rosenmai et al. 2014). Although the authors did not examine the mechanism of action of progesterone up-regulation, previous work suggested a direct inhibition of the CYP17 (cytochrome P450 17A1) lyase reaction, independent of ER action (Zhang et al. 2011). Thus, BPA analogs may have additional disruptive effects that have not been detected with BPA.

## Discussion

Although relatively few studies have examined the hormonal actions of BPS and BPF (especially *in vivo*), the *in vitro* literature indicates that BPS and BPF have actions and potencies similar to those of BPA and supports the biological plausibility of their hormonal activity *in vivo.* This is not surprising because BPF and BPS are structural analogs of BPA and thus mechanisms of action would be expected to be similar. For example, BPF showed cumulative, possibly synergistic, actions *in vivo* when co-administered with an androgen (Yamasaki et al. 2003), and BPA has also been shown to have these types of effects when combined with other hormones or xenoestrogens (Kang et al. 2002; Silva et al. 2002). Particularly interesting is the fact that BPS seems to have actions on nongenomic signaling similar to those of BPA (Viñas and Watson 2013a, 2013b). BPA is sometimes called a “weak” estrogen because of its relatively weak binding/activation of the nuclear receptors compared with E_2_, although this is not always the case (Table 3; Kitamura et al. 2005; Perez et al. 1998; Pisapia et al. 2012). However, when the nongenomic estrogenic activity of BPA was measured, it was comparable, if not more potent, than E_2_. This potent, nongenomic estrogenic activity of BPA has been described in several experimental models (Alonso-Magdalena et al. 2008, 2012; Viñas and Watson 2013a, 2013b; Watson et al. 2014). The potency of BPS in a nongenomic signaling assay was similar to that of BPA. In femtomolar to picomolar concentrations, BPS induced membrane ERα-mediated pathways and actions: MAPK (mitogen-activated protein kinase) signaling, cell proliferation, and activation of caspase 8 (Viñas and Watson 2013a, 2013b). These rapid, nongenomic pathways are important for optimal cell function, mediating proliferation and apoptosis (Viñas and Watson 2013a, 2013b), as well as other actions such as pancreatic cell function (Alonso-Magdalena et al. 2008) and estrogen-mediated brain function and behavior (Laredo et al. 2014; Moenter and Chu 2012).

BPS and BPF had potencies in the same order of magnitude as BPA. The issue of potency is complicated because of the fact that lowest observed effect levels depend on end point, receptor type, pathway, tissues, windows of exposure, and so on. In general, BPS was slightly less potent than BPA. The average BPF potency was similar to BPA, with a fairly wide range of potencies. However, the implications of these differences are not clear. In regard to potency, it is not known whether a compound that is, for example, half as potent as BPA *in vitro* would have half the effect *in vivo*, especially because very little is known about the exposure and metabolism of BPS and BPF. Further, even if potencies of BPS and BPF are slightly less than that of BPA, it is unclear if these compounds are safer; many scientists have advocated a “no-threshold” approach to endocrine disruption because thresholds may change during development or may be very difficult to assess (Munn and Goumenou 2013).

The metabolism and biological fate of BPS and BPF have not been well studied, but *in vitro* and *in vivo* experiments indicated that BPF metabolism and distribution are similar to those of BPA. *In vitro,* BPA was metabolized by human and rat hepatic cells to many different metabolites, including non-bioactive sulfate and glucuronide conjugates (Cabaton et al. 2008; Dumont et al. 2011). *In vivo*, BPF administered to pregnant rats via gavage resulted in the excretion of BPF and several metabolites in the urine, including the nonactive sulfate-conjugated BPF. Active BPF was also distributed to many tissues, including the uterus, placenta, amniotic fluid, and fetuses. The ratio of the active parent compound to the metabolites/conjugates was similar to that of BPA (Cabaton et al. 2006; Vandenberg et al. 2013b). The primary route of excretion for BPF appeared to be through the sulfatase conjugate, rather than the glucuronide conjugate (as with BPA). Cabaton et al. (2006) suggested that this may be due to the fact that BPF glucuronide may be more easily deconjugated to its bioactive state and reabsorbed in large quantities, which also appears to occur with BPA (Vandenberg et al. 2013b). No studies have assessed the metabolism of BPS or the bioactivity of the metabolites. Studies determining the metabolism of BPS and the bioactivity of metabolites from BPF and BPS are warranted.

The body of literature on the *in vivo* effects of BPS and BPF is scant, but it points to these chemicals as endocrine disruptors and reproductive toxicants. BPS induced uterine growth in rodents (indicative of estrogenic action) and disrupted reproduction in fish (Ji et al. 2013; Naderi et al. 2014; Yamasaki et al. 2004), and BPF also had uterotropic (estrogenic) effects in female rodents and gonadotropic (androgenic) effects in male rodents (Higashihara et al. 2007; Stroheker et al. 2003, 2004; Yamasaki et al. 2004). Although most of the *in vitro* data support estrogenic, and to some extent, antiandrogenic, actions of BPS and BPF (Table 3), one *in vitro* study showed that BPS has androgenic activity similar to BPA (Molina-Molina et al. 2013). Thus, the *in vitro* data support the *in vivo* observations of hormonal and endocrine disruptive activity of these compounds.

Concern over the endocrine-disruptive effects of BPA has resulted in hundreds of laboratory studies, including *in vitro* (Wetherill et al. 2007) and *in vivo* (Richter et al. 2007b; Vandenberg 2014b) studies, identifying estrogenic and other effects. Although some regulators have rejected this body of literature because of a lack of standardized protocols, reviews of these studies have indicated strong methodologies and stringent laboratory practices, often of higher quality than studies employing Good Laboratory Practices (Myers et al. 2009). Many *in vivo* BPA studies have demonstrated adverse outcomes at “low” (i.e., environmentally or physiologically relevant) doses (Vandenberg 2014a; Vandenberg et al. 2012). Many studies also report that BPA has a nonlinear, or nonmonotonic, dose–response curve. Nonmonotonic dose responses are indicative of an endocrine-mediated response and are consistent with natural hormone responses (Vandenberg 2014b; Vandenberg et al. 2012, 2013a; Zoeller et al. 2012). Further, nearly 100 human studies described the relationship between BPA and several endocrine-related health impacts on reproduction, neurodevelopment, thyroid function, and metabolic health (Rochester 2013). Although epidemiological studies are less controlled than laboratory animal experiments, making it difficult to show causation, they are important indicators of potential health effects (Diamanti-Kandarakis et al. 2009; Zoeller et al. 2012). Further, although BPA is quickly metabolized and excreted from the body [with a half life of about 6 hr (Dekant and Völkel 2008)], the fact that it is found in almost all humans sampled at any one time suggests the ubiquitous and constant nature of BPA exposure (Vandenberg et al. 2010), which is disconcerting in light of the animal and human evidence of health effects. Many researchers have raised concern over this overwhelming evidence and have called for stricter regulation of BPA (Vandenberg et al. 2009, 2012). Although this concern has prompted BPA to be phased out of certain products (Food and Drug Administration 2012), the structural analog replacements may not be any safer.

Because BPS and BPF appear to have metabolism, potencies, and mechanisms of action *in vitro* similar to BPA, including hormonal actions beyond that of BPA, they may pose similar potential health hazards as BPA. Therefore, when evaluating the safety of compounds for consumer use, it may be prudent to consider entire classes instead of individual compounds. In addition, as other researchers have suggested (Viñas and Watson 2013a), future research efforts should focus on designing chemical substitutes that do not have biological or hormonal activity similar to those of BPA. Further, this review demonstrates that systematic reviews may be useful in the process of conducting safety evaluations of chemical classes. The use of the bisphenol class of compounds as replacements for BPA in consumer products with high human contact should be implemented with caution.
